# Antioxidant Capacities and Total Phenolic Contents Enhancement with Acute Gamma Irradiation in *Curcuma alismatifolia* (Zingiberaceae) Leaves

**DOI:** 10.3390/ijms150713077

**Published:** 2014-07-23

**Authors:** Sima Taheri, Thohirah Lee Abdullah, Ehsan Karimi, Ehsan Oskoueian, Mahdi Ebrahimi

**Affiliations:** 1 Department of Crop Science, Faculty of Agriculture, Universiti Putra Malaysia, 43400 Serdang, Selangor, Malaysia; E-Mail: ehsan_b_karimi@yahoo.com; 2 Institute of Tropical Agriculture, Universiti Putra Malaysia, 43400 Serdang, Selangor, Malaysia; E-Mail: ehs424@yahoo.com; 3 Department of Veterinary Preclinical Sciences, Faculty of Veterinary Medicine, Universiti Putra Malaysia, 43400 Serdang, Selangor, Malaysia; E-Mail: mehdiebrahimii@gmail.com; 4 Agriculture Biotechnology Research Institute of Iran (ABRII)-East and North-East Branch, P.O.B. 91735/844 Mashhad, Iran

**Keywords:** antioxidant activity, bioactive compounds, *Curcuma alismatifoli*, gamma irradiation

## Abstract

The present study was conducted in order to assess the effect of various doses of acute gamma irradiation (0, 10, 15, and 20 Gy) on the improvement of bioactive compounds and their antioxidant properties of *Curcuma alismatifolia* var. *Sweet pink*. The high performance liquid chromatography (HPLC) and gas chromatography (GC) analysis uncovered that various types of phenolic, flavonoid compounds, and fatty acids gradually altered in response to radiation doses. On the other hand, antioxidant activities determined by 1,1-Diphenyl-2-picryl-hydrazyl (DPPH), ferric reduction, antioxidant power (FRAP), and 2,2-azino-bis-3-ethylbenzothiazoline-6-sulfonic acid (ABTS) radical scavenging assay showed a higher irradiation level significantly increased the antioxidant properties. This study revealed an efficient effect of varying levels of gamma radiation, based on the pharmaceutical demand to enhance the accumulation and distribution of bioactive compounds such as phenolic and flavonoid compounds, fatty acids, as well as their antioxidant activities in the leaves of *C. alismatifolia* var. *Sweet pink*.

## 1. Introduction

Bioactive compounds often accumulate in the plant in small quantities and sometimes in specific cells [1]. Among them some are known as phenolic, flavonoids, and essential oils, which possess a wide range of biological activities such as antioxidant, anti-inflammatory, anti-aging, anti-bacterial, anti-tumor, and other functions [2,3].

Flavonoids and phenolic acids are the most important groups of secondary metabolites in plants that consider as good sources of natural antioxidants in human diets [4]. These compounds are able to scavenge free superoxide radicals, reduce the risk of cancer, and protect biological systems against the deleterious effects of oxidative incidents on macromolecules, such as lipids, proteins, carbohydrates, and DNA. Based on previous studies flavonoids and phenolic acids were introduced as an antioxidant [5,6].

Antioxidants are compounds known to slow or delay lipid oxidation. Suppressive antioxidants can separate free radicals or single oxygen before any significant oxidant occurrence. However, chain-breaking antioxidants delay or slow the oxidative processes after they start up [7,8].

Consumer demand for healthier products containing less synthetic additives is driving research efforts to seek out alternative sources of natural antioxidants. Phenolic and flavonoid compounds are natural antioxidant that found in plants and they are attracting a great deal of attention due to increasing evidence suggesting that they may prevent chronic conditions, such as cancer, atherosclerosis, and neurological diseases [9].

The genus *Curcuma* from Zingiberaceae family originated from the Indo-Malayan Region [10] with a wide-spread distribution in the tropics of Asia to Africa and Australia.

*C. alismatifolia* is a monocotyledonous perennial, originating from tropical and subtropical areas of Northern Thailand and Cambodia [11].

One of the most challenging pursuits in the realm of pharmaceutical and medical sciences is to investigate for latest and more potent drugs with fewer toxic effects and completely reversible. Much of these features can easily find from the natural compounds of plants [12]. Several years, gamma irradiation has been considered as a rapid and new method to enhance the qualitative and quantitative characters of many plants. Gamma irradiation has been widely used in biology and medicine in terms of biological effects of low dose stimulation to high-dose inhibition [13]. Several studies have shown that relatively low-doses ionizing irradiation on photosynthetic microorganisms and plants accelerated cell growth, cell proliferation, germination rate, enzyme activity, crop yields, as well as stress resistance [14]. Therefore, this experiment was conducted to analyze the bioactive compounds, such as phenolic, flavonoid, and fatty acids, using HPLC and GC, respectively. Furthermore, their antioxidant activities were evaluated under different treatment of the acute gamma irradiation in the leaves of *C. alismatifolia* var. *sweet pink*.

## 2. Results and Discussion

### 2.1. Total Phenolics (TP) and Flavonoids (TF) Content

The results indicated that the accumulation of TP and TF in the plant leaves was considerably affected by different levels of acute gamma irradiation and it had a significant (*p* < 0.05) effect on TP and TF of irradiated groups (Table 1). The overall results demonstrated that leaves under 20 Gy of acute gamma irradiation exhibited higher content of TP and TF with values of 3.15 ± 1.73 (GAE)/g DW and 2.87 ± 0.31 rutin/g DW compared to Control with respective values of 2.08 ± 0.12 (GAE)/g DW and 1.61 ± 0.48 rutin/g DW, respectively. Previous studies demonstrated that various forms of irradiation influenced the phenolics and flavonoids content. Gamma irradiation (10 KGy) increased phenolic acid content in cinnamon and clove while phenolic content in nutmeg did not change [15,16]. Variyar *et al.* [16] indicated that the free phenolic (aglycone) content of the soybean samples treated with gamma irradiation at levels ranging from 0.5 to 5 kGy increased. The increment of TP and TF content under different levels of gamma irradiation could be ascribed to the release of these compounds from glycosidic forms and the degradation of larger compounds into smaller ones by gamma irradiation [17].

**Table 1:** Total phenolics and flavonoids content in the leaves of *C. alismatifolia* var. *sweet pink* under different dose levels of acute gamma irradiation (Mean ± SEM; *n* = 3).

Dose	Phenolic Content ^1^	Flavonoid Content ^2^
Control	2.08 ± 0.12 ^a^	1.61 ± 0.48 ^a^
10 (Gy)	2.11 ± 1.25 ^a^	1.88 ± 1.82 ^a^
15 (Gy)	2.76 ± 0.32 ^b^	2.09 ± 0.57 ^b^
20 (Gy)	3.15 ± 1.73 ^c^	2.87 ± 0.31 ^c^

^1^ mg gallic acid equivalent/g DW; ^2^ mg rutin equivalent/g DW; ^a,b,c^ Means with the same letter between different columns are not significantly different at *p* < 0.05.

### 2.2. Profiling of Phenolic and Flavonoid Compounds Using HPLC

**Figure 1:**
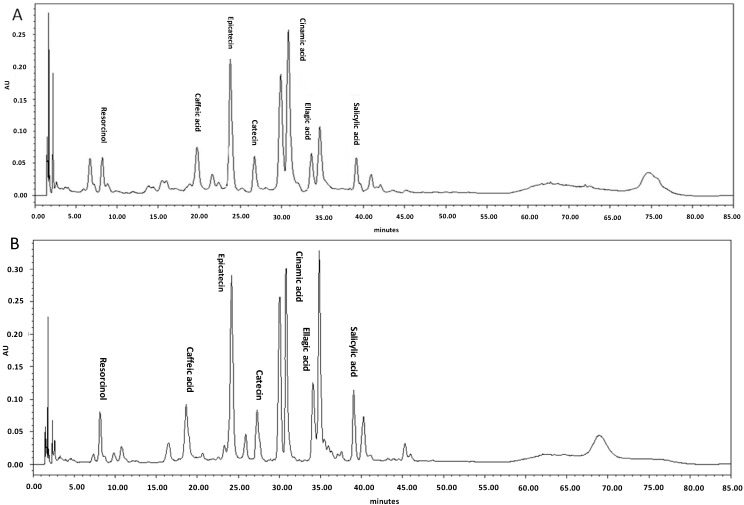
(**A**) The HPLC chromatogram of phenolic compounds in the non-treated leaves of *C. alismatifolia* var. *sweet pink*; (**B**) The HPLC chromatogram of phenolic compounds in the leaves of *C. alismatifolia* var. *Sweet pink* under 20 Gy acute gamma irradiation. Compound identification as labeled (concentration: 1 mg/mL).

HPLC is currently the method of choice to accurately determine both the composition and the absolute concentration of the secondary metabolites of a sample [18]. The phenolic and flavonoid compounds were identified based on their conservation times and quantified according to respective standard calibration curves. The HPLC chromatogram revealed that cinnamic acid and rutin were the main phenolic and flavonoid compounds with values of 1015 μg/g DW and 1032.7 μg/g DW in the leaves of studied *C. alismatifolia* var. *Sweet pink*, respectively (Table 2 and Table 3). It is apparent that phenolic and flavonoid accumulation and partitioning was considerably affected by differentlevels of acute gamma irradiation. Meanwhile, increasing dose levels of acute gamma irradiation from 5 to 20 Gy resulted in enhancement of various types of phenolic and flavonoid compounds (Table 2 and Table 3). Previous researchers demonstrated that the phenolic acid content increased by gamma irradiation (10 kGy) treatment with cinnamon and clove while phenolic content in nutmeg did not change [15]. The HPLC chromatogram in Figure 1A,B shows the phenolic compounds in the leaves of *C. alismatifolia* var. *Sweet Pink* under different levels of acute gamma irradiation (0 and 20 Gy) as an instance.

**Table 2:** Concentration of different phenolic compounds in the leaves of *C. alismatifolia* var. *sweet pink* under different dose levels of acute gamma irradiation (Mean ± SEM; *n* = 3).

Phenolic Contents (µg/g Dry Sample)								
Dose	Gallic Acid	Salicylic Acid	Caffeic Acid	Catechin	Epicatechin	Cinnamic Acid	Ellagic Acid	Resorcinol
Control	ND	406.2 ± 37.72 ^d^	125.2 ± 7.663 ^d^	212.9 ± 15.61 ^d^	856.4 ± 57.05 ^d^	1015.4 ± 76.15 ^d^	182.6 ± 12.12 ^d^	195.9 ± 16.71 ^d^
10 (Gy)	ND	595.5 ± 51.43 ^c^	181.6 ± 9.12 ^c^	231.2 ± 17.04 ^c^	795.2 ± 45.32 ^c^	1033.1 ± 67.18 ^c^	204.7 ± 16.76 ^c^	227.5 ± 18.33 ^c^
15 (Gy)	ND	688.1 ± 56.11 ^b^	203.7 ± 15.01 ^b^	255.6 ± 21.12 ^a,b^	877.1 ± 36.72 ^b^	1052.7 ± 56.26 ^b^	235.1 ± 18.27 ^b^	253.5 ± 21.22 ^b^
20 (Gy)	ND	785.3 ± 34.25 ^a^	227.2 ± 13.02 ^a^	269.1 ± 23.51 ^a^	928.3 ± 78.94 ^a^	1081.5 ± 89.35 ^a^	269.3 ± 21.03 ^a^	282.1 ± 23.67 ^a^

ND: not detected; ^a^^,b,c,^^d^ Means within columns with different superscript are significantly different at *p* < 0.05.

**Table 3:** Concentration of different flavonoids compounds in the leaves of *C. alismatifolia* var. *sweet pink* under different dose levels of acute gamma irradiation (Mean ± SEM; *n* = 3).

Flavonoid Contents (µg/g Dry Sample)					
Dose	Rutin	Naringin	Apigenin	Quercetin	Myricetin
Control	1032.7 ± 67.05 ^d^	271.5 ± 17.01 ^d^	ND	964.1 ± 76.05 ^d^	166.1 ± 9.51 ^d^
10 (Gy)	1286.5 ± 89.03 ^c^	355.1 ± 21.01 ^c^	ND	1025.8 ± 86.07 ^c^	225.6 ± 14.42 ^c^
15 (Gy)	1545.5 ± 111.09 ^b^	482.9 ± 34.03 ^b^	ND	1131.3 ± 75.12 ^b^	282.5 ± 16.02 ^b^
20 (Gy)	1704.7 ± 123.05 ^a^	564.1 ± 43.05 ^a^	ND	1292.4 ± 91.05 ^a^	351.1 ± 21.02 ^a^

ND: not detected; ^a^^,b,c,^^d^ Means within columns with different superscript are significantly different at *p* < 0.05.

### 2.3. Fatty Acid Composition of Irradiated Leaves

The fatty acid composition of the *C. alismatifolia* leaves with different gamma irradiation treatment levels has been presented in Table 4. The proportion of leaves fatty acids having 18 carbons was quite consistent across the four treatment levels, ranged from 49.29% to 58.15% (Table 4). Mean concentrations of C18:0, C18:1n-9, C18:2n-6, and C18:3n-3 were 5.22%, 11.05%, 16.59%, and 20.76%, respectively. On the other hand, C18:3n-3 increased in a linear manner with increasing the irradiation intensity. The different levels of irradiation showed significant (*p* < 0.05) effects on C18:3n-3 in the leaves. It has been reported that a number of intracellular constituents, including pigments [19], amino acids [20], and fatty acids [21], which could be responsible for radio-resistance. Byun *et al*. [22] reported non-significant changes in the fatty acid profile of soybean seeds with different intensity of gamma irradiation in contrast Štajner *et al.* [23] established that doses up to 10 kGy caused insignificant changes in total lipids, fatty acid composition, peroxide value, and trans fatty acid content of soybean. The gamma irradiation for *C. alismatifolia* leaves showed a significant increase in omega-3 fatty acid as the intensity of gamma irradiation increased.

**Table 4:** Leaves Fatty acid composition of *C. alismatifolia* var. *Sweet Pink* under different dose levels of acute gamma irradiation (Mean ± SEM; *n* = 3).

	Radiation Doses (Gy)			
Fatty Acids	Control (0)	10 (Gy)	15 (Gy)	20 (Gy)
C12:0	1.17 ± 0.06	2.02 ± 0.11	1.04 ± 0.04	1.15 ± 0.05
C14:0	18.61 ± 1.02 ^ab^	21.48 ± 1.18 ^a^	16.49 ± 0.91 ^b^	15.84 ± 0.87 ^b^
C14:1	0.67 ± 0.04	0.47 ± 0.03	0.41 ± 0.02	0.46 ± 0.03
C15:0	3.46 ± 0.19	3.00 ± 0.16	2.79 ± 0.15	2.28 ± 0.13
C15:1	7.72 ± 0.42	7.29 ± 0.40	6.25 ± 0.34	4.66 ± 0.26
C16:0	23.90 ± 1.31	23.34 ± 1.28	24.76 ± 1.36	24.46 ± 1.34
C16:1	1.76 ± 0.10	2.30 ± 0.13	2.09 ± 0.11	2.03 ± 0.11
C17:0	0.66 ± 0.04	0.68 ± 0.04	0.70 ± 0.04	0.64 ± 0.04
C17:1	0.55 ± 0.03	0.47 ± 0.03	0.50 ± 0.03	0.66 ± 0.04
C18:0	6.36 ± 0.35	5.16 ± 0.28	4.46 ± 0.25	4.90 ± 0.27
C18:1n-9	10.65 ± 0.59	10.77 ± 0.59	12.20 ± 0.67	10.60 ± 0.58
C18:2n-6	16.73 ± 0.92	14.06 ± 0.77	18.06 ± 0.99	17.54 ± 0.96
C18:3n-3	18.10 ± 0.99 ^b^	19.29 ± 1.06 ^b^	20.58 ± 1.13 ^ab^	25.10 ± 1.38 ^a^
^1^ Total saturated fatty acid	54.16 ± 2.98 ^a^	55.69 ± 3.06 ^a^	50.24 ± 2.76 ^b^	49.26 ± 2.71 ^b^
^2^ Total monounsaturated fatty acid	19.59 ± 1.08	19.00 ± 1.04	19.36 ± 1.06	16.39 ± 0.90
^3^ Total polyunsaturated fatty acid	34.82 ± 1.91 ^b^	33.36 ± 1.83 ^b^	38.64 ± 2.12 ^ab^	42.65 ± 2.34 ^a^
^4^ Total n-3 PUFA	18.10 ± 0.99 ^b^	19.29 ± 1.06 ^b^	20.58 ± 1.13 ^ab^	25.10 ± 1.38 ^a^
^5^ Total n-6 PUFA	16.73 ± 0.92	14.06 ± 0.77	18.06 ± 0.99	17.54 ± 0.96

^1^ Total saturated fatty acid = sum of C12:0 + C14:0 + C15:0 + C16:0 + C17:0 + C18:0; ^2^ Total monounsaturated fatty acid = sum of C14:1 + C15:1 + C16:1 + C17:1 + C18:1n-9; ^3^ Total polyunsaturated fatty acid = C18:2n-6 + C18:3n-3; ^4^ Total n-6PUFA = sum of C18:2n-6; ^5^ Total n-3PUFA = sum of C18:3n-3; ^a,b,ab^ Means within rows with different superscript are significantly different at *p* < 0.05.

### 2.4. Antioxidant Activity (DPPH, FRAP and ABTS Scavenging)

**Figure 2:**
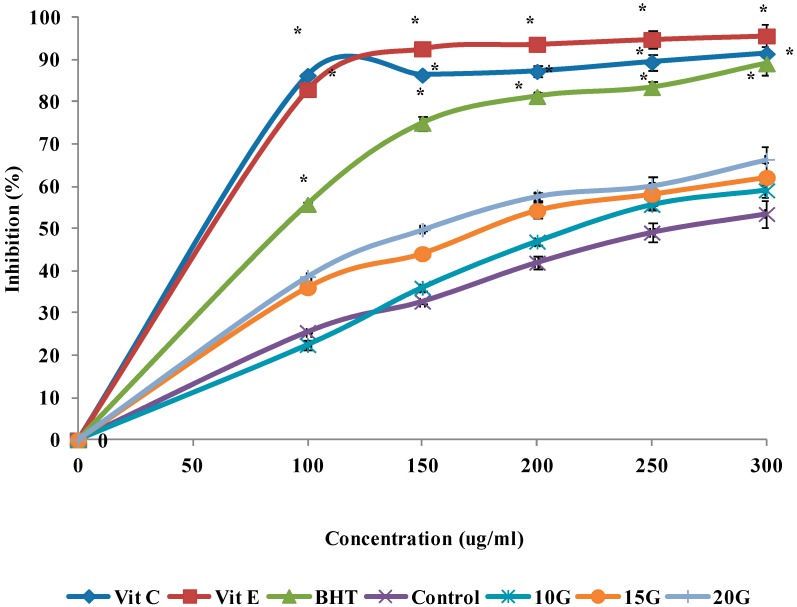
Effect of gamma irradiation on the free radical scavenging activity of methanolic extracts and standards at different concentrations. Values are means ± SE. Each value represents the mean of three replications. * Significance different from Control group at the same concentration. Significance was determined at *p* < 0.05.

**Figure 3:**
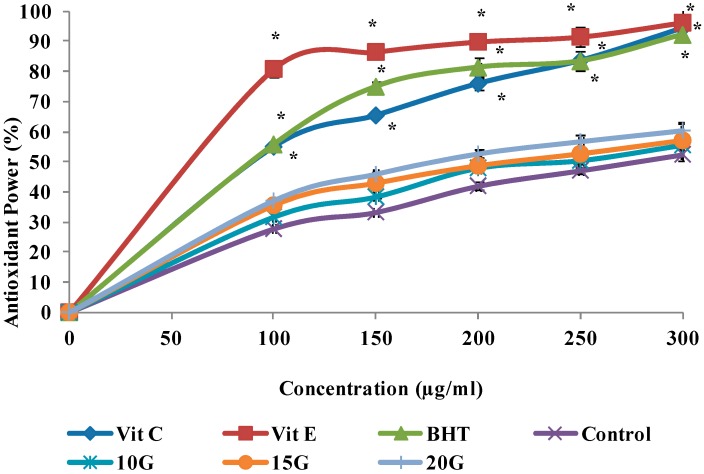
Effect of gamma irradiation on the ferric reduction antioxidant power activity of methanolic extracts and standards at different concentrations. Values are means ± SE. Each value represents the mean of three replications. * Significance different from Control group at the same concentration. Significance was determined at *p* < 0.05.

**Figure 4:**
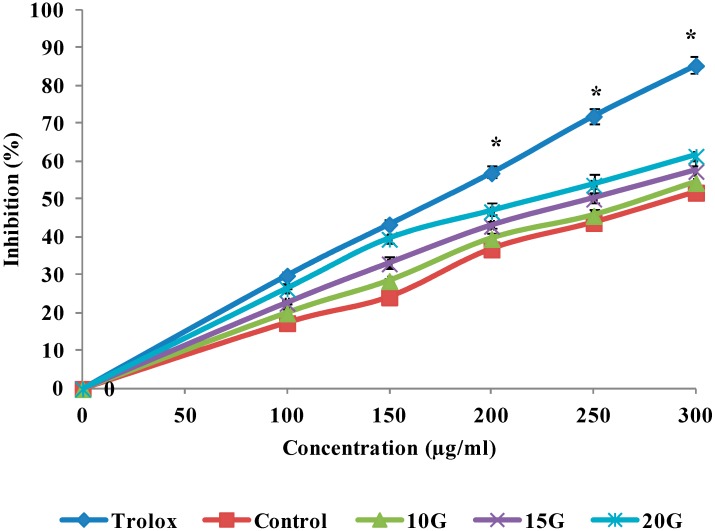
Effect of gamma irradiation on the ABTS radical scavenging activities of methanolic extracts and positive control at different concentrations. Values are means ± SE. Each value represents the mean of three replications. * Significance different from Control group at the same concentration. Significance was determined at *p* < 0.05.

*Curcuma* species plants have received much attention, since they produce many beneficial compounds that are useful in the food industry as herbs, flavoring and in the medical industries as an antioxidant and antimicrobial agents. The antioxidant activities of extracts obtained from *C. alismatifolia* leaves under different gamma irradiation levels in the reactions with DPPH, FRAP, and ABTS assay, illustrated in Figure 2, Figure 3 and Figure 4, respectively. The antioxidant activities of the extracts generally as the irradiation dose increased and this stimulation reached to its maximum at a dose level of 20 Gy. The IC_50_ value of the treated and standard leaf extracts presented in Table 5. Radiation induced oxidative injury by speeding up free radical production in living systems. Radiation stimulates damage to DNA, cell membrane and protein structure (Battino *et al.*) [24]. The initial damage induced by ionizing radiation is altered in enzymatic repair processes [25]. Several studies showed that the *C. alismatifolia* leaves have antioxidant activities [26,27]. Other leaves from *Curcuma* spices such as *Curcuma zanthorrhiza and Curcuma longa* also showed strong antioxidant activities [28,29]. It was previously shown that gamma irradiation significantly influenced the cell metabolism and protein synthesis in plant meristem cells [30]. Lee *et al.* [31] reported an increase in total phenols and total flavonoids in irradiated plants. Such increase in total flavonoids and phenols can be due to the degradation of larger phenolic compounds into smaller compounds or release of phenolic compounds from glycosidic components of irradiated leaves as explained by Harrison and Were [17]. Vegetative traits and flowering development showed significant changes, when gamma irradiation at different doses applied for *C. alismatifolia* var. *Sweet Pink* [32]. The previous study by *Quiles et al.* [33] indicted that the best-known antioxidant mechanism of curcuma and its components is their capacity to eliminate reactive oxygen species, such as hydroxyl radical, superoxide radical, singlet oxygen, and NO. They results showed the other points of view regarding the antioxidant mechanism of curcuma extract by protection of the endogenous antioxidants from oxidative damage. In irradiated plants, the leaf length, leaf width, inflorescence length, the number of true flowers, the number of pink bracts, number of shoots, and plant height, decreased significantly (*p* < 0.05) as the radiation dose increased.

**Table 5:** The IC_50_ values of extracts and standards in DPPH, FRAP and ABTS scavenging activities (Mean ± SEM; *n* = 3).

	IC_50_ (µg/mL)		
Samples	Free Radical Scavenging Activity	Total Antioxidant Activity	ABTS Scavenging Activity
Control	260.7 ± 1.29 ^a^	278.1 ± 1.42 ^a^	288.1 ± 1.35 ^a^
10 (Gy)3	218.5 ± 0.72 ^b^	244.8 ± 1.56 ^b^	275.8 ± 0.96 ^b^
15 (Gy)	179.3 ± 1.03 ^c^	218.4 ± 2.05 ^c^	248.9 ± 1.12 ^c^
20 (Gy)	152.6 ± 0.85 ^d^	180.7 ± 1.66 ^d^	225.2 ± 1.25 ^d^
Vitamin C	58.1 ± 1.47 ^f^	90.9 ± 2.11 ^e^	ND
Vitamin E	60.3 ± 3.04 ^f^	61.88 ± 1.86 ^f^	ND
BHT	89.7 ± 2.43 ^e^	89.7 ± 1.37 ^e^	ND
Trolox	ND	ND	174.47 ± 012 ^e^

ND: not detected; ^a,b,c,d,e,f^ Means within columns with different superscript are significantly different at *p* < 0.05; DPPH: 1,1-Diphenyl-2-picryl-hydrazyl; FRAP: ferric reduction, antioxidant power; ABTS: 2,2-azino-bis-3-ethylbenzothiazoline-6-sulfonic acid.

## 3. Experimental

### 3.1. Plant Material

The plant materials used in this study were the rhizomes of *C. alismatifolia* var. *sweet pink* (SK 2052/12), measuring of about 2.0–2.5 cm with 4–7 storage root. Rhizomes were provided from a *Curcuma* nursery (Ubonrat) in Doisaket District, Chiang Mai 50220, Thailand.

### 3.2. Planting Media and Preparation of Rhizomes for Acute Gamma Irradiation

The non-irradiatedrhizomes were treated with a fungicide (Benlate^®^, DuPont Co., Johnston, IA, USA) before planting in a mixture of topsoil: cocopeat: rice husk at the ratio of 1:2:1. Four rhizomes planted per 25 cm pot. Twenty days after planting, the rhizomes in the sprouting bud stage are dug out from the soil, washed with tap water, dried at room temperature, and wrapped in an aluminum foil (15 × 15) cm^2^.

### 3.3. Irradiation Treatment

Irradiation of the rhizomes was conducted in the Faculty of Science and Technology, University Kebangsaan Malaysia (UKM) using a Gamma cell 220 Excel Irradiator (MDS Nordion, Ottawa, ON, Canada). The source of gamma rays was Cobalt 60 (^60^Co). Prepared rhizomes in sprouting bud stage were acutely irradiated at dose levels of 10, 15, 20 Gy at room temperature (25 ± 1 °C). Twenty rhizomes irradiated at each dose level and 20 rhizomes were considered as control. The irradiated rhizomes were planted in 25 cm pots containing growth media consisting of topsoil:cocopeat:rice husk at the ratio of 1:2:1. The experiment was conducted in Greenhouse No. 1, Field 2, Faculty of Agriculture, Universiti Putra Malaysia (UPM), Malaysia. The experiment was designed as randomized complete block design (RCBD) with five blocks and four replications. The pots were watered manually to saturation once every 2 days, starting after planting of the bulbs. Ovide (Malathion^®^ 50%, Shanghai AgroChina Chemical Co., Ltd., Shanghai, China) and Benomyl (Benlate^®^ 50%,Wilmington, NC, USA), were sprayed weekly to prevent the incidence of unwanted pests and diseases. The soil was fertilized with a NPK (15-15-15) once a month at the rate of 5 g per pot [34]. The plants were grown in greenhouse for three months (from April to July), and the leaves of treated and non-treated individuals were harvested before flowering starts. The leaf of treated and non-treated samples were freeze-dried, ground to a powder using mortar and pestle and were kept in −80 °C for further experiments.

### 3.4. Extract Preparation

The leaf extraction procedure was followed according to the Crozier *et al.* [35] using methanol. The freeze-dried leaves (2 g) were soaked into a 100 mL conical using 40 mL of 80% (*v*/*v*) aqueous methanol with the addition of 10 mL hydrochloridric acid (6 M). The mixture was then refluxed at 90 °C for 2 h and filtrated by No. 1 filter paper (Whatman, Camlab Inc., Cambridge, UK) followed by evaporation of the filtrate using a vacuumed Rotary Evaporator (Rotavapor® R-215, Buchi Inc., Uster, Switzerland). The final extracts were dissolved in HPLC grade methanol for determination of flavonoids and phenols.

### 3.5. Total Phenols and Flavonoids Determination

The total phenols and flavonoids were determined according to Ismail *et al.* [36]. For total phenol determination, briefly 0.5 mL of each methanolic extract, 2 mL of 7.5% sodium carbonate, and 2.5 mL Folin-Ciocalteu reagent, were mixed together. The mixture was then vortex and incubated for 90 min at room temperature. The absorbance was read using a visible spectrophotometer (Novaspec II Visible Spectrophotometer, Pharmacia Biotech, Cambridge, UK) at 765 NM. The total phenol results were expressed as mg gallic acid equivalents (GAE)/g dry weight (DW). For total flavonoid compounds 0.1 mL of methanolic extracts was added to 0.3 mL sodium nitrite (5%) and incubated for 5 min at room temperature, then 0.3 mL 10% (*w*/*v*) AlCl_3_ and 2 mL 1 N NaOH was added and the total volume was made up to 5 mL with distilled water. The absorbance was measured at 510 nm by using visible spectrophotometer at 510 nm. The results were expressed as mg rutin equivalents/g DW.

### 3.6. Evaluation of Phenolic and Flavonoid Compounds

The phenolic and flavonoid compounds of samples quantitatively measured by reversed-phase high performance liquid chromatography (HPLC) technique based on Crozier *et al.* [35]. The standards for phenolic compounds were ellagic acid, salicylic acid, gallic acid, catechin, epicatechin, caffeic acid, cinnamic acid, and resorcinol. The standard for flavonoid compounds were naringin, apigenin, rutin, quercetin, and myricetin. The sample extract was injected on an HPLC Agilent-1200 series instrument equipped with an auto sampler and column (Intersil ODS-3 5 um 4.6 × 150 mm Gl Science Inc., CA, USA), pump and UV-Vis photodiode array (DAD) detector. Two solvents including acetonitrile and deionized water were used for mobile phase. The pH of deionized water was adjusted to be at 2.5. The flavonoid compounds were identified at 350 nm and phenolic and iso-flavonoid compounds were determined at 280 nm.

### 3.7. Fatty Acid Profiles

*C. alismatifolia* leaves total fatty acids were extracted according to the method of Folch *et al.* [37] with some modifications by Ebrahimi *et al.* [38], using chloroforms: methanol 2:1 (*v*/*v*) which contained butylated hydroxytoluene to prevent the oxidation during fatty acid extraction. Extracted fatty acids Transmethylated to the fatty acid methyl esters (FAME) using KOH in methanol and boron trifluoride (BF_3_). The FAME were separated using gas liquid chromatography (Agilent 7890A), using a Supelco SP 2560 capillary column of 100 m × 0.25 mm ID × 0.2 µm film thickness (Supelco, Inc., Bellefonte, PA, USA). One microliter was injected into the gas chromatography, equipped with an injector and a flame ionization detector. The nitrogen was the carrier gas at a flow rate of 1.2 mL/min. The split ratio was 1:20. The temperature of injector was 250 °C and the detector temperature was 270 °C. The column temperature program started runs at 150 °C, for 2 min, warmed to 158 °C at 1 °C/min, held for 28 min, warmed to 220 °C at 1 °C/min, and then held for 20 min. A reference standard (C4-C24 methyl esters; Sigma-Aldrich, Inc., St. Louis, MS, USA), was used to determine correction factors for the determination of individual fatty acid composition. The data are expressed as g/100 g of detecting total fatty acids.

### 3.8. Antioxidant Activity

#### 3.8.1. DPPH Free Radical Scavenging Activity

The DPPH of the extracts were determined by Gulcin *et al.*’s [39] method. The activities of DPPH were expressed as percentage of inhibition and calculated by following equation according to Yen and Chen [39].

% inhibition of DPPH activity = [(A_0_ − A_1_)/A_0_)] × 100% (1)

where A_0_ was the absorbance value of the control or blank sample and A1 was the absorbance value of the test sample. A curve of % inhibition or % scavenging effect against sample concentrations was plotted and the concentration of the sample required for 50% inhibition was determined. The value for each of the test sample was shown as the inhibition curve at 50%. BHT, α-tocopherol and vitamin C were utilized as standard antioxidants.

#### 3.8.2. Ferric Reducing Antioxidant Power (FRAP)

The FRAP property of the extracts was determined using a method as described by Yen and Chen [40]. The test was completed in triplicate. BHT, α-tocopherol and vitamin C were utilized as standard antioxidants.

#### 3.8.3. ABTS Radical Cation-Scavenging

The ABTS was evaluated by the method of Giao *et al*. [41]. ABTS was dissolved in water; to a 7 mm concentration. ABTS radical cation (ABTS^•^^+^) was produced by reacting ABTS stock solution with 2.45 mM K_2_S_2_O_8_ and allowing the mixture to stand at room temperature (dark place) overnight before utilization.

### 3.9. Statistical Analysis

All data are presented as means (±SEM) of at least three replicates (*n* = 3). The total phenolic and flavonoid contents, fatty acid, DPPH, and FRAP were analyzed using analysis of variance (ANOVA) with the Statistical Analysis System (SAS) Version 9.1 (SAS Institute, Cary, NC, USA). Significant differences among means from triplicate analyses (*p* < 0.05) were determined by Duncan’s Multiple Range Test. The level of significance was set at *p* < 0.05 for all statistical tests.

## 4. Conclusions

Gamma irradiation has been generally used in biology and medicine regarding biological activity effects. In plant breeding projects, mutagenic treatments with low negative physiological impacts and suitable genetic effects are desirable. Consequently, we utilized more powerful and effective doses of gamma irradiation (10, 15, and 20 Gy) of which, especially, the 20 Gy dose was viable to influence morphological attributes of examining *C. alismatifolia* variety. In addition, results from previous studies, showed higher percentage of plant growth mutation and getting wanted mutants at 20 Gy of acute gamma irradiation. In addition, from this experiment, it is inferred that radiation dose up to 20 Gy can enhance the quality and amount of bioactive compounds, including phenolic and flavonoid, in addition to the improvement of scavenging activity in *C. alismatifolia* var. *Sweet pink* leaves.
